# Relevance of G-quadruplex structures to pharmacogenetics

**DOI:** 10.3389/fphar.2014.00160

**Published:** 2014-07-08

**Authors:** Simone L. Cree, Martin A. Kennedy

**Affiliations:** Department of Pathology, Carney Centre for Pharmacogenomics, University of OtagoChristchurch, New Zealand

**Keywords:** drug targets, G-quadruplex (G4), gene expression, gene regulation, secondary structure

## Abstract

G-quadruplexes are non-canonical secondary structures formed within nucleic acids that are involved in modulating cellular processes such as replication, gene regulation, recombination and epigenetics. Within genes, there is mounting evidence of G-quadruplex involvement in transcriptional and post transcriptional regulation. We report the presence of potential G-quadruplex motifs within relevant sites of some important pharmacogenes and discuss the possible implications of this on the function and expression of these genes. Appreciating the location and potential functions of these motifs may be of value when considering the impacts of some pharmacogenetic variants. G-quadruplexes are also the focus of drug development efforts in oncology and we highlight the broader pharmacological implications of treatment strategies that may target G-quadruplexes.

## Introduction

The desire to implement pharmacogenetic testing as a means to improve drug safety and treatment efficacy has led to intense scrutiny of the functional and clinical relevance of variation in pertinent genes (Sim et al., 2013). Over 300 human genes are recognized to be involved in the absorption, distribution, metabolism, and excretion of drugs, as well as encoding protein targets for therapeutics (Sangkuhl et al., 2008; Madian et al., 2012). Extensive cataloging of variants within these genes has been carried out (Nelson et al., 2012), with the 1000 Genome Project providing a global view of such variants (Durbin et al., 2010). In addition, genome wide association studies (GWAS) and high throughput gene expression analyses are implicating new genes in drug response phenotypes (Daly, 2012; Madian et al., 2012; Wheeler and Dolan, 2012). Our ability to interpret the clinical significance of this growing catalog of gene variants depends on a fuller appreciation of functional genomic features (Sadee et al., 2011). Here we seek to highlight the importance of a genomic feature called the G-quadruplex that has been implicated in many critical cellular functions, and which may be of considerable interest to those working in pharmacogenomics.

**Figure 1:**
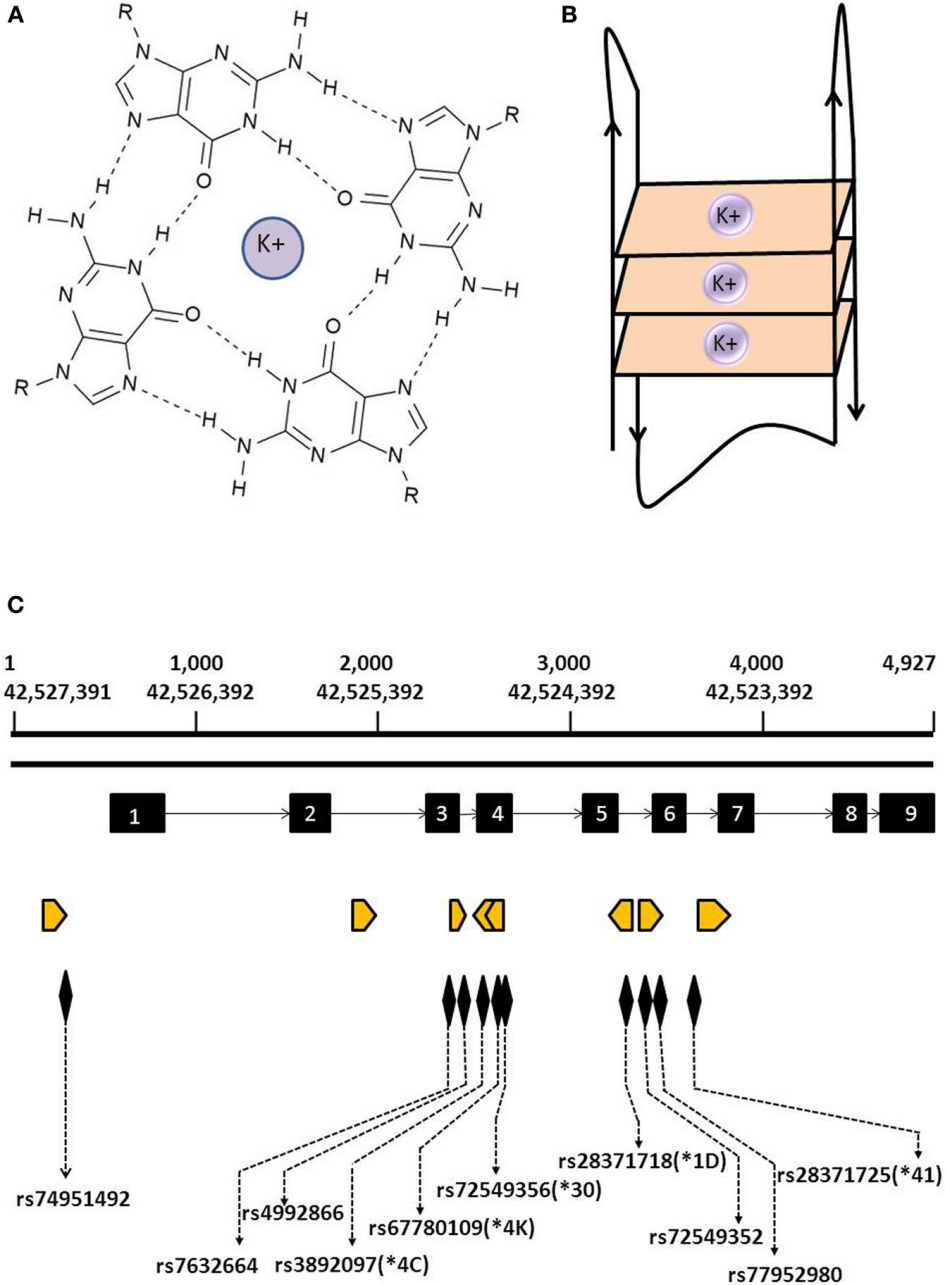
**Structure of G4 and location of predicted G4s within the *CYP2D6* gene. (A)** Guanine tetrad formed by the association of four guanine bases via Hoogsten hydrogen bonds in a coplanar arrangement and stabilized by a potassium ion. **(B)** Schematic representation of a G-quadruplex formed by the stacking of the three guanine tetrads from a single strand of DNA. **(C)** Location of predicted G4s in the *CYP2D6* gene. Orange arrowheads indicate location and orientation of predicted G4s; exons are depicted by black blocks. Also indicated are the locations of SNPs and CYP2D6^*^ alleles found within predicted G4s.

G-quadruplexes (G4s) are stable secondary structures found in G rich nucleic acids wherein guanine bases associate via Hoogsteen hydrogen bonds to form tetrads (Figure 1A) that stack in a planar arrangement with a stabilizing monovalent cation occupying a central position of the cavity (Sen and Gilbert, 1988; Sundquist and Klug, 1989). G4s can form in one strand (Figure 1B) or from multiple strands where sequences intervening G tracts of two or more guanines exist as loops of various sizes (Burge et al., 2006). The stability and topology of G4 are influenced by several factors including length and sequence composition, strand stoichiometry, and orientation, nature of the binding cation and loop size between G tracts with longer tracts and small sized loops contributing to more stable structures (Bugaut and Balasubramanian, 2008; Guedin et al., 2010). The existence and topology of G4s can be studied *in vitro* by methods such as circular dichroism, nuclear magnetic resonance, and X-ray crystallography whereas fluorescence imaging, small molecule and protein pull downs, ChIP-Seq and ChIP-chip experiments can be designed to detect G4s in cellular DNA (Di Antonio et al., 2012b).

The human genome contains more than 375,000 predicted G4 forming sequences (Huppert and Balasubramanian, 2005) with clusters of them occurring in biologically important regions such as telomeres, promoters, 5′-untranslated regions (UTRs), 3′UTRs, replication origins, exons and introns. G4s are implicated in maintenance of chromosomal ends, transcription, translation, DNA replication, alternative splicing, recombination, and epigenetic stability (Maizels and Gray, 2013). It is not yet clear how often putative G4 motifs actually give rise to G4 structures *in vivo*, although this process is expected to be tightly regulated within different cell types. Evidence for G4 formation *in vivo* has come from the identification of G4 interacting cellular proteins and the ability of small molecules that bind G4 to affect transcription, translation and replication (Rodriguez, 2012; Biffi et al., 2013; Valton et al., 2014). Furthermore, the location and composition of G4s are conserved in human populations, implying that they play an important role in cellular processes (Eddy and Maizels, 2008).

G4 formation in the promoters of proto-oncogenes has been linked with repression of transcription; most likely due to polymerase pausing at the site of G4 formation. Addition of small molecule ligands was found to stabilize G4s in the promoters of the proto-oncogene *RET* (Shin et al., 2014) and the gene encoding tumor angiogenesis factor *VEGF* (Salvati et al., 2014) resulting in altered protein expression. Likewise, RNA G4s in the 5′UTR of the matrix metalloproteinase (*MT3-MMP*), estrogen receptor (*ESR1*), apoptotic regulator (*BCL2*), telomere shelterin protein (*TRF2*) and several other proteins have been reported to negatively modulate translation in gene-reporter expression studies (Morris and Basu, 2009; Bugaut and Balasubramanian, 2012). RNA G4s have also been shown to play a role in alternative splicing and expression patterns of genes *Bcl-xL* (Hai et al., 2008) and *FMR1*(Didiot et al., 2008) while modulation of G4 formation in the third intron of the tumor supressor gene *TP53* was recently shown to result in varied expression of the p53 transcript (Marcel et al., 2011).

Owing to the abundance of predicted G4s in the telomeric regions of the genome and in promoters of many oncogenes, including *MYC*, G4 represent an appealing and novel target for small-molecule chemotherapeutic development (Verma et al., 2009; Neidle, 2010). Inhibition of *MYC* transcription was achieved by exposing HeLa S3 cells to the small molecule TMPyP4, which binds to its promoter G4, providing proof of principle for the targeting of regulatory G4 structures of oncogenes as a novel therapeutic strategy (Siddiqui-Jain et al., 2002). Likewise, ongoing research is directed toward promoter G4 mediated transcriptional repression of proto-oncogenes such as the receptor tyrosine kinase *KIT*, the small GTPase *KRAS*, and more recently of an RNA G4 in the 5′ UTR region of the small GTPase gene *NRAS* (Heinrich et al., 2003; Bugaut et al., 2010).

Given the apparent significance of G4s, we have analyzed the occurrence of potential G-quadruplex forming sequence within “Very Important Pharmacogenes” (VIP) (Whirl-Carrillo et al., 2012) using web based G4 prediction algorithms Quadparser (Wong et al., 2010) and QGRSH (Kikin et al., 2006). The VIP is a manually curated list of genes with well described pharmacogenetic relationships which includes genes that encode drug targets, metabolic enzymes as well as drug transporters. It is by no means an exclusive listing of all known pharmacogenes, but it includes many of the best understood and widely studied pharmacogenes. Our analysis indicates that predicted G4 are found in the promoters, 5′UTRs, 3′UTRs, exons, and introns of pharmacogenes. The position and location of predicted G4 within the VIP suggests the potential for G4 structures to impact on pharmacogene expression (Supplementary Table 1).

## G4s and the regulation of pharmacogenes

### Promoter G4s function as transcriptional regulatory elements

Studies focused on interaction of promoter G4s within the cell have revealed a role in transcriptional regulation. The proto-oncogene *MYC* which is overexpressed in more than 80% of solid tumors contains a G4 forming region in the nuclease hypersensitive element of its promoter (Siddiqui-Jain et al., 2002). The nuclear protein nucleolin promotes G4 formation leading to transcriptional repression (Gonzalez et al., 2009). Conversely, transcriptional activation of this gene is induced by the G4-unwinding activity of the nucleoside diphosphate kinase NM23H2 protein (Postel et al., 1993) and poly ADP-ribose polymerase (Fekete et al., 2012). Palumbo et al. (2008) found that multiple GGA repeats within the *C-MYB* promoter form a G4 that acts as a transcriptional repressor by interacting with Myc-associated Zn finger proteins. Likewise, transcriptional repression of the human *PDGFB* gene was observed by ligand-mediated stabilization of its promoter G4 (Qin et al., 2010). In addition, it appears that the transcriptional regulatory protein SP1 can bind to G4 structures with high affinity, implying a role for transcription factor binding sites to form G4 as a key determinant for regulation of some genes (Raiber et al., 2012).

More than 40% of human gene promoters contain at least one predicted G4 (Huppert and Balasubramanian, 2007); in line with this our analysis of the VIP list (Whirl-Carrillo et al., 2012) indicates the occurrence of at least one promoter predicted G4 in 38% of these genes (Supplementary Table 1). Among these are genes encoding important metabolic enzymes (*CYP2D6, CYP2J2, G6PD, TPMT*), receptor proteins (*ADRB1, ADRB2, DRD2, VDR*), folate transporter (*SLC19A1*) and proteins involved in potassium voltage-gated and sodium channels (*KCNH2, SCN5A*). Presence of predicted G4 in the promoters of these pharmacogenes is suggestive of transcriptional regulation at the promoter G4 site. Mutations or polymorphisms affecting these predicted G4 could alter stability of secondary structures impacting gene expression and activity, and this may be important when considering the regulatory potential of genetic variants. This type of an effect was demonstrated by the recent observation at rs2255888 of the lipoxygenase gene *ALOX15* promoter implicated in pathogenesis of atherosclerosis. Using biophysical and *in vitro* studies the G but not the A allele at this position was found to be involved in the DNA secondary structure formation (although this did not appear to be a G4), affecting binding of the cytoskeleton protein vimentin and resulting in altered transcriptional regulation (Samanta et al., 2012).

### Role of RNA G4s in post transcriptional regulation

Evidence has emerged for the role of G4s not only in transcription but also in post transcriptional regulation, as G4s can readily form in RNA molecules (Biffi et al., 2014). RNA G4s occur within the 5′UTR, exons, introns and 3′UTRs where they regulate translation, alternative splicing and expression patterns (Marcel et al., 2011; Bugaut and Balasubramanian, 2012; Endoh et al., 2013; Murat et al., 2014). G4s formed in the 5′UTR were found to play a role in translational down-regulation of genes with cap-dependent translation (Morris and Basu, 2009; Bugaut and Balasubramanian, 2012) whereas they have been shown to mediate internal ribosome entry site translation initiation in cap-independent translation (Bonnal et al., 2003; Morris et al., 2010).

Our analysis revealed G4 motifs in the 5′UTRs of the pharmacogenes *G6PD, GSTP1, KCNH2*, and *PTGIS* (Supplementary Table 1), suggesting these motifs have the potential to affect translational efficiencies and modulate protein expression levels. Detailed *in silico, in vitro* and cellular analysis of SNPs among 5′UTR predicted RNA G4s provide further evidence for G4 involvement in translation (Beaudoin and Perreault, 2010). Beaudoin and Perreault (2010) demonstrated that a G to C substitution within the 5′UTR RNA G4 of *AASDHPPT* (L-aminoadipate-semialdehyde dehydrogenase-phosphopantetheinyl transferase) restricted G4 formation by favoring stem loop structures, which led to increased translation efficiency of a reporter gene. This example also illustrates the potential significance of SNPs within G4 of 5′UTRs in causing differential gene expression among individuals.

Our investigation of G4 motifs in the VIP gene set also reveals that 20% of the genes queried contain a predicted G4 in their 3′UTR. This includes *ACE, ADRB1, CYP2A6, G6PD, MTHFR, SLC19A1*, and *VDR* (Supplementary Table 1). The presence of predicted G4s in the 3′UTR implies a role for RNA G4s in other stages of RNA metabolism. A 3′UTR in the *PIM1* proto-oncogene was reported to reduce translation efficiency (Arora and Suess, 2011). Recent findings indicate that 3′UTR G4s can also regulate gene expression by increasing the efficiencies of alternate polyadenylation sites leading to expression of shorter transcripts, and eluding of the miRNA regulatory network (Beaudoin and Perreault, 2013).

### Potential G4s in the *CYP2D6* gene

The highly polymorphic *CYP2D6* gene is of particular pharmacogenetic interest owing to its implications for metabolism of a variety of drugs (Becker and Leeder, 2010). We detected a total of eight predicted G4s in this gene (Supplementary Table 1; Figure 1C) with one present 437 bp upstream from the transcriptional start site in the promoter region which could potentially influence transcriptional regulation. We also observed predicted G4 within introns 2, 3, 5, and 6 in an orientation such that they would occur in nuclear pre-mRNA transcripts (Figure 1C), with possible relevance to RNA processing. Notably, some of the CYP2D6^*^ alleles involved in poor and rapid metabolizer phenotypes are situated within predicted G4s. Among these are CYP2D6^*^1D (rs28371718), CYP2D6^*^4K (rs67780109), CYP2D6^*^4C (rs3892097), CYP2D6^*^30 (rs72549356) and CYP2D6^*^41 (rs28371725). Furthermore, dbSNPs rs76326664 (A/G), rs77952980 (A/G), rs4992866 (C/T) are located within predicted G4 in *CYP2D6* introns where they may affect G4 formation and stability. In addition to SNPs, mutations such as insertions, deletions, and gene rearrangements all have the ability to exert effects on G4 stability and hence perturb their role in transcriptional or translational regulation.

## G4s as drug targets

### G4 interacting ligands in cancer therapy

The large amount of research into G4 ligand induced gene regulation over the last decade has led to several G4 targeting compounds that are currently under investigation as potential chemotherapeutic drugs. G4 targeting anticancer drugs evaluated for some well-studied proto-oncogenes alongside their advantage and disadvantages are listed in Table 1. The first line of ligands such as the tri-substituted acridines targeted the terminal quartet present at the top and bottom of the G4 structure (Mergny et al., 2002). Platinum-derived complexes have been shown to preferentially target the *MYC* G4 structure over duplex DNA (Wang et al., 2013). The fluoroquinone derivative quarfloxin was the first G4-interacting compound to reach Phase II clinical trials, with potential for the treatment of neuroendocrine tumors (Drygin et al., 2009). Its ability to strongly interact with parallel G4 structures has been studied in detail within the cellular context. Although treatment with quarfloxin revealed the absence of organ or genotoxicity, it led to the disruption of interaction between nucleolin and ribosomal RNA G4 with consequent inhibition of Pol I driven transcription and redistribution of nucleolin within the cell, limiting its bioavailability (Balasubramanian et al., 2011).

**Table 1:** **List of G4 binding anticancer drugs tested**.

**G-quadruplex interacting drug**	**Advantage**	**Disadvantage**	**References**
Trisubstituted acridines	Targets large aromatic surface at top and bottom of G4, effective telomerase inhibitor	Non-specific interactions	Mergny et al., 2002
Quindoline and Berberine	Antiproliferative, Myc downregulation in cancer cell lines	Binds duplex DNA, non-specific interactions	Ou et al., 2007
TyMPYP4	Transcriptional repression of *MYC*	Non-specific interactions	Siddiqui-Jain et al., 2002
Trisubstituted isoalloxazines	Stabilizes *KIT* promoter G4, transcriptional repression	Non-specific interactions	Bejugam et al., 2007
Telomestatin	Antitelomerase and anti-Myc activity	Non-specific interactions	Kim et al., 2002
Naphthalene diimide	Dose dependant cell arrest of mutated *KIT* cell lines	Non-specific interactions	Gunaratnam et al., 2009
Platinum derived complexes	Targets G4 of *c-MYC* over duplex	Non-specific interactions if any are yet to be determined	Wang et al., 2013
Quarfloxin	Interacts with parallel G4	Limited bioavailability	Drygin et al., 2009

As chemotherapeutic drugs that target regulatory G4 enter and move through clinical trials it may be prudent to consider whether binding of drugs to non-target G4s could impact on expression of genes that are important in metabolism of other chemotherapeutic drugs and xenobiotics. We have identified predicted G4 in a number of pharmacogenes relevant to the treatment of cancer (Supplementary Table 1) which could conceivably be impacted by such off target effects of G4 drugs. For example, if a G4 targeting drug also targets the predicted G4 in the promoter of *CYP2D6* gene that is involved in transcriptional repression, it may result in reduced efficacy of other drugs that are metabolized by CYP2D6, like tamoxifen (Province et al., 2014). Similar situations could arise if the drug targeted G4s within the *TPMT* or *UGT1A1* gene whose products are involved in metabolism of thiopurine drugs (Weinshilboum, 2001), and irinotecan (Wang et al., 2011) respectively.

### More widespread opportunities for G4 based therapeutics?

Although much effort has been directed toward understanding G4s in proto-oncogenes and more recently cardiovascular genes (Zhou et al., 2013), there has been little consideration of the potential significance of G4s in many of the genes that impact on responses to treatment of these and other diseases. Among these are the G-protein coupled receptors, ADRB1 and ADRB2 involved in mediating heart rate, contractibility, bronchodilator response and cardiomyopathy (Sandilands and O'shaughnessy, 2005; Lymperopoulos and Bathgate, 2012). It is worth considering whether G4 formation in these and other important pharmacogenes could represent valid targets for modification of drug metabolism using targeted therapeutics.

We detected putative G4 in the promoter and 5′UTR of the *TPMT* gene (Supplementary Table 1), and it may be of value to investigate the formation of G4s and the role they may play in the regulation of this gene product as it is involved in the treatment of autoimmune diseases, inflammatory bowel disease, lupus, transplantation, and acute lymphoblastic leukemia (Lennard, 2002). We also described variant trinucleotide repeats within the promoter of *TPMT* from two patients with ultra-high activity of this enzyme, which showed increased transcriptional activity in reporter gene assays (Roberts et al., 2008). These GCC repeat arrays may be capable of forming G4 structures, and different forms of these could conceivably contribute to different levels of transcriptional activity.

In addition to *CYP2D6*, discussed above, other predicted G4 containing pharmacogenes include *UGT1A1*, involved in bilirubin metabolism and degradation and removal of xenobiotic waste (Tukey and Strassburg, 2000); *CYP2A6*, best known for its role in conversion of nicotine to cotinine (Benowitz et al., 2006); and *MTHFR*, important in folate and methionine biosynthesis (Marini et al., 2008) as well as *de novo* purine biosynthesis (Jongbloet et al., 2008) and DNA methylation. Determining the formation of G4 structures at these sites and establishing whether this is of pharmacogenetic significance is important as there may be merit in exploring novel strategies for targeting G4 mediated regulation in these and other genes.

### Future of G4 drug targeting

The quest to identify and devise G4 binding ligands has garnered much attention in the last few years. Development of small molecules with high specificity and affinity for a particular G4 could also be achieved by using structure-based design methods and screening against virtual compound libraries (Ma et al., 2011). Investigation of the alkyl derivative of TMPyP4 (TMpyP4-C14), led to the observation that TMpyP4-C14 could efficiently enter cells and preferentially localize into the cytoplasm with binding to G4 structures in the 5′UTR of *KRAS* mRNA, resulting in 90% down regulation of *KRAS* protein expression in pancreatic cancer cells (Xodo et al., 2008). Small molecules like the pyridine-2,6-bis-quinolinodicarboxamide derivative and its variant have been shown to specifically decrease translation efficiency by stabilizing a G4 in the 5′UTR of the *NRAS* proto-oncogene (Bugaut and Balasubramanian, 2012). *In situ* click chemistry is another promising approach aimed at enhancing the interaction specificity of a small molecule G4 ligand to its corresponding nucleic acid structure (Di Antonio et al., 2012a). Virtual screening approaches on the other hand have identified novel G4 ligands (Alcaro et al., 2013; Gonzalez et al., 2013). Another approach to molecules with selective affinity for specific G4 is to generate specific antibodies (Fernando et al., 2009). Huppert (2007) presented the idea of adding functional specificity to the G4 binding ligand by tagging it with sequence specific complementarity to its neighboring DNA. The biological activity of these compounds depends on the structural interaction of G4 small molecule ligand complexes to disrupt normal regulatory processes that may involve the targeted G4 structure.

G4 aptamers that bind to and block clinically relevant proteins are the converse situation, and the prototypical molecule of this class is the thrombin binding G4 aptamer which inhibits thrombin catalyzed clot formation (Nagatoishi et al., 2011). Similarly, HIV DNA integration into the genome was shown to be inhibited by binding of G4 aptamers to the HIV integrase protein (Do et al., 2011). Finally, G4 decoys could be explored in treating patients that harbor G4 alleviating SNPs in clinically relevant genes. This strategy was shown to successfully mediate apoptosis in HeLa and T24 urinary bladder cancer cells expressing the hyper activated HRAS protein. G4 decoys designed to mimic the promoter G4s of the *HRAS* proto-oncogene functioned as transcriptional repressors (Membrino et al., 2011). Introduction of G4 decoys also resulted in effective inhibition of *KRAS* and tumor growth arrest in pancreatic cancer cells (Cogoi et al., 2013).

## Conclusion

Among the many DNA and RNA structures that have been described, G4s have gained increasing prominence because of their implication in so many fundamental cellular, evolutionary and genomic processes (Wu and Brosh, 2010; Maizels and Gray, 2013). Although there is an increased understanding of the role played by G4s in a number of cellular processes the structural intricacies of G4 motifs associated with specific function remain to be explored. Small molecule based G4 binding ligands have proved successful in regulating transcription and expression of proto-oncogenes, providing a novel approach to the treatment of cancerous cells (Siddiqui-Jain et al., 2002; Gunaratnam et al., 2009). Our survey revealed several important pharmacogenes that harbor one or more predicted G4 in relevant sites that may contribute to regulation and expression. Investigating the role of these predicted G4s may also provide evidence for alternative targets through which specific regulation of pharmacogenes could be achieved in situations where this may confer clinical benefit. While G4s located in regulatory regions of pharmacogenes may provide opportunities for pharmacokinetic modulation by small molecules that target G4, they also represent a risk for off-target regulatory effects of chemotherapeutics designed to target regulatory G4 in oncogenes. For example, tamoxifen and codeine are both prodrugs that are activated by CYP2D6. If a small molecule designed to target an oncogene G4 also acted on the G4 sites in *CYP2D6*, reducing expression of this metabolic enzyme, unexpected effects on the efficacy of tamoxifen or codeine could occur. Alternatively, if the chemotherapeutic drug itself was a substrate of CYP2D6, then concurrent modulation of CYP2D6 via off-target G4 binding could markedly affect the pharmacokinetic behavior of the drug.

Finally, G4 structures are also vulnerable to modulation by mutation or polymorphism, so our analysis has the potential to assign regulatory significance to newly discovered pharmacogenetic variants that may affect function by impacting on such structural features. Because pharmacogenes, like oncogenes and other classes of genes, have the potential to be regulated by formation of G4s within either genomic DNA or RNA transcripts, it is our hope that the knowledge of G4 structures and consideration of their potential to be modified may provide useful insights in the field of pharmacogenomics.

### Conflict of interest statement

The authors declare that the research was conducted in the absence of any commercial or financial relationships that could be construed as a potential conflict of interest.
