# Supplementary material for: Relevance of G-quadruplex structures to pharmacogenetics
Source: Front Pharmacol. 2014 Jul 8;5:160. doi: 10.3389/fphar.2014.00160 (PMC4085647; doi:10.3389/fphar.2014.00160)
Supplement: Supplementary file 1 [file Table1.PDF]

Supplementary Material

Relevance of G-quadruplex structures to pharmacogenetics

Cree, S.L [1], Kennedy, M.A [1]*

[1] Carney Centre for Pharmacogenomics, Department of Pathology, University of Otago, Christchurch, New Zealand

*** Correspondence:** Martin A Kennedy, Carney Centre for Pharmacogenomics, Department of Pathology, University of Otago, P.O. Box 4345, Christchurch, New Zealand
Martin.kennedy@otago.ac.nz

1. Supplementary Tables

Supplementary Table 1. Occurrence of putative G4s in promoters and UTRs of PharmGKB VIP genes

Gene Symbol	Description	G4s predicted by Quadparser*		G4s predicted by QGRS Mapper* with G scores ≥ 30	
		**Number**	**Location**	**Number**	**Location**
ACE	Angiotensin-converting enzyme, somatic isoform precursor (EC 3.4.15.1)<br>Diseases: Angioedema, Cough, Hypertension	6<br>2	Promoter<br>3’UTR	7<br>1	Promoter<br>3’UTR
ADRB1	Beta-1 adrenergic receptor<br>Diseases: Alzheimers, angina, Cardiovascular diseases, Hypertension, Depression, Obesity, Sleep apnoea, Ischemia	3<br>1<br>1	Promoter<br>5’UTR<br>3’UTR	5<br>1	Promoter<br>3’UTR
ADRB2	Beta-2 adrenergic receptor<br>Diseases: Acute coronary syndrome, Asthma, Coronary artery disease, Heart failure, Hypertension	1	Promoter	2	Promoter
AHR	Aryl hydrocarbon receptor precursor<br>Metabolism: Xenobiotics	1	Promoter	4	Promoter
ALOX5	Arachidonate 5-lipoxygenase (EC 1.13.11.34)<br>Diseases: Aspirin-induced asthma, Colonic neoplasms, Coronary artery disease	3<br>1	Promoter<br>5’UTR	3<br>1	Promoter<br>5’UTR
CYP2A6	Cytochrome P450 2A6 (EC 1.14.14.1)<br>Metabolism: Acetaminophen, Artemisinin, Caffeine, Carbamazepine, Cyclophosphimide, Fluoropyrimidine, Ifosfamide, Nicotine, Phenytoin, Valproic acid, Zidovudine	1	3’UTR	2	3’UTR

CYP2D6	Cytochrome P450 2D6 (EC 1.14.14.1)<br>Metabolism: Acetaminophen, Atorvastatin, Benzodiazepine, Celecoxib, Citalopram, Clomipramine, Codeine &morphine,Doxepin, Fluoxetine, Fluvastatin, Gefitinib, Imatinib, Imipramine, Nevirapine, Phenytoin, Statin, Tamoxifen, Tramadol	1	Promoter	1	Promoter
CYP2J2	Cytochrome P450 2J2 (EC 1.14.14.1)<br>Diseases: Asthma, Ischemia, Coronary artery Disease, Coronary disease, Coronary stenosis, Hypertension	1	Promoter	1	Promoter
DRD2	D(2) dopamine receptor<br>Diseases: Schizophrenia	1	Promoter	2	Promoter
G6PD	Glucose-6-phosphate 1-dehydrogenase<br>Diseases: Anemia, Congenital Nonspherocytic, Arteriosclerosis, Cardiovas cular Diseases, Diabetes Mellitus, Favism, HIV, Hyperbilirubinemia, Jaundice, Metabolic SyndromeX, Methemoglobinemia, Neoplasms, Obesity	2<br>2<br>2	Promoter<br>5'UTR<br>3'UTR	2<br>2	Promoter<br>3'UTR
GSTP1	Glutathione S-transferase P (EC 2.5.1.18)<br>Metabolism: Acetaminophen, Busulfan, Etoposide, Platinum	1<br>1	Promoter<br>5'UTR	1<br>1	5'UTR<br>3'UTR
KCNH2	Potassium voltage-gated channel subfamily H member 2<br>Diseases: Acquired Long QT Syndrome (aLQTS), Atrial fibrillation, congenital long QT syndrome, Long QT Syndrome, Shortened QT interval, Tachycardia, Ventricular Torsades de Pointes	5<br>2	Promoter<br>5'UTR	4<br>5<br>1	Promoter<br>5'UTR<br>3'UTR
MTHFR	Methylenetetrahydrofolate reductase (EC 1.5.1.20).<br>Disease: Alzheimers, Arthritis, Rheumatoid Cardiovascular diseases, Cleft Lip, Cleft Palate, Down syndrome, Hyperhomocysteinemia, Neoplasms, Neural tube defects, Pre-eclampsia	1<br>1<br>2	Promoter<br>5'UTR<br>3'UTR	1	Promoter
NQO1	NAD(P)H dehydrogenase [quinone] 1 (EC 1.6.5.2)<br>Diseases: Leukemia, Lung Neoplasms [7]	1	Promoter	1	Promoter
NR1I2	Orphan nuclear receptor PXR (Pregnane X receptor)<br>Diseases: Colitis, Crohn disease, Endometrial neoplasms, Inflammatory bowel diseases, Osteosarcoma, Ovarian neoplasms, Prostatic neoplasms	1	Promoter	1	Promoter
PTGIS	Prostacyclin synthase (EC 5.3.99.4)<br>Diseases: Cerebral infarction, Hypertension, Myocardial Infarction	7<br>1<br>1	Promoter<br>5'UTR<br>3'UTR	10<br>1<br>1	Promoter<br>5'UTR<br>3'UTR

SCN5A	Sodium channel protein type 5 subunit alpha<br>Diseases: Brugada syndrome, Heart block, Long QT syndrome, Sick sinus syndrome, Sudden infant death, Ventricular fibrillation	1	3'UTR	3	Promoter
SLC19A1	Folate transporter 1<br>Diseases: Congenital Heart defect, Thrombosis	1<br>1<br>7	Promoter<br>5'UTR<br>3'UTR	2<br>1<br>8	Promoter<br>5'UTR<br>3'UTR
TPMT	Thiopurine S-methyltransferase (EC 2.1.1.67)<br>Diseases: Myelosupression<br>Metabolism: Thiopurines	4<br>1	Promoter<br>5'UTR	6<br>1	Promoter<br>5'UTR
VDR	Vitamin D3 receptor (VDR)<br>Diseases: Acquired Immunodeficiency Syndrome, Asthma, Breast neoplasms, Diabetes Mellitus, Myocardial Infarction, Osteoporosis, Prostatic Neoplasms, Psoriasis, Rickets, Tuberculosis<br>Metabolism: Etoposide	2	3'UTR	2<br>1<br>1	Promoter<br>5'UTR<br>3'UTR
UGT1A1	UDP glucuronosyltransferase 1A1;<br>Diseases: Crigler-Najjar Syndrome, Gilbert disease, Neonatal jaundice	1	Promoter	1	Promoter

***Quadparser predicted G4s with motif "G_3N_{1-7} G_3N_{1-7} G_3N_{1-7} G_3N_{1-7}" whereas QGRS Mapper was used to predict G4s with motif "G_2N_{1-36} G_2N_{1-36} G_2N_{1-36} G_2N_{1-36}" and G scores ≥ 30**
